# Potential pitfalls of mass spectrometry to uncover mutations in childhood soft tissue sarcoma: A report from the Children’s Oncology Group

**DOI:** 10.1038/srep33429

**Published:** 2016-09-19

**Authors:** Lin Xu, Raphael A. Wilson, Theodore W. Laetsch, Dwight Oliver, Sheri L. Spunt, Douglas S. Hawkins, Stephen X. Skapek

**Affiliations:** 1 Department of Pediatrics Division of Hematology/Oncology, and the Harold C. Simmons Cancer Center, University of Texas Southwestern Medical Center, and the Pauline Allen Gill Center for Cancer and Blood Disorders, Children’s Medical Center, Dallas, TX, USA; 2 Department of Pathology, and the Harold C. Simmons Cancer Center, University of Texas Southwestern Medical Center, Dallas, TX, USA; 3 Department of Pediatrics, Division of Hematology/Oncology, Stanford University School of Medicine, Palo Alto, CA, USA; 4 Division of Hematology/Oncology, Seattle Children’s Hospital, Fred Hutchison Cancer Research Center, University of Washington, Seattle, WA, USA.

## Abstract

Mass spectrometry-based methods have been widely applied – often as the sole method – to detect mutations in human cancer specimens. We applied this approach to 52 childhood soft tissue sarcoma specimens in an attempt to identify potentially actionable mutations. This analysis revealed that 25% of the specimens harbored high-confidence calls for mutated alleles, including a mutation encoding FLT3^I836M^ that was called in four cases. Given the surprisingly high frequency and unusual nature of some of the mutant alleles, we carried out ultra-deep next generation sequencing to confirm them. We confirmed only three mutations, which encoded NRAS^A18T^, JAK3^V722I^ and MET^R970C^ in three specimens. Beyond highlighting those mutations, our findings demonstrate potential pitfalls of primarily utilizing a mass spectrometry-based approach to broadly screen for DNA sequence variants in archived, clinical-grade tumor specimens. Duplicate mass spectrometric analyses and confirmatory next generation sequencing can help diminish false positive calls, but this does not ameliorate potential false negatives due in part to evaluating a limited panel of sequence variants.

Sequenom MassARRAY (Sequenom, San Diego, CA) is one of most popular high-throughput technologies to detect mutations in DNA samples. Compared to next generation sequencing (NGS) platforms, this mass spectrometry-based assay purports to provide rigorous genetic characterization with lower cost^1^,^2^, making it ideal for genome-wide association studies (GWAS)^3^,^4^ and clinical diagnosis^5^,^6^. Mutations detected by this approach are generally considered to be highly reliable. Indeed, we used an in-house text-mining algorithm in a search of PubMed entries to select 200 publications in which MassARRAY findings were not subjected to further validation. When closely scrutinizing 60 that we chose to represent a spectrum of peer-reviewed journals, we confirmed the lack of secondary validation in all and the lack of replicate testing by MassARRAY in the majority (Table S1). The approaches in those reports may have been justified by extensive preliminary validation of the assay to detect a limited number of variant alleles in each laboratory. Here, we utilized MassARRAY in a different way: to broadly screen for sequence variants in DNA extracted from formalin-fixed, paraffin-embedded (FFPE) childhood tumor specimens, with plans for secondary validation of variant alleles by using next-generation sequencing.

We studied 52 FFPE tumor specimens representing 18 different types of pediatric sarcoma or related soft tissue neoplasm (Table S2) collected as part of the Children’s Oncology Group (COG) D9902 Soft Tissue Sarcoma Biology and Banking Study. The de-identified DNA specimens were processed according to the MassARRAY guidelines, and the OncoCarta v1.0 and v3.0 panels were employed to interrogate 365 actionable mutations in 33 cancer-related genes in all 52 specimens. The MassARRAY Typer software identified 15 high confidence mutations in 13 cases, as well as a larger number of lower confidence calls. The high confidence calls included *FLT3*^*I836M*^ (called in four cases) and two different mutant *BRAF* and *STK11* alleles in three and two cases, respectively (Table S3). We conducted targeted ultra-deep sequencing (averaged 1368× after removing PCR duplicates) to verify 14 of the mutations in those cases with sufficient remaining DNA. We were only able to confirm 3 of the 14 mutations tested (*NRAS*^*A18T*^, *JAK3*^*V722I*^ and *MET*^*R970C*^) (Table S3). The three validated mutations were found in cases with high or low DNA concentration, A_260/280_ ratio, and mutant allele frequency (Fig. 1). We calculated the false discovery rate (FDR), which is the proportion of positive test results that are false discoveries. In our studies, mass spectrometric detection of this panel of variants in DNA extracted from FFPE material was associated with a high rate of false positives (FDR = 79%). Although we point out that the FDR appeared worse when the mutant allele frequency was 30% or less (FDR = 92%), the small number of cases limits our ability to conclude that the high FDR might be due to specific factors like low DNA concentration or chemical contaminants that might interfere with mass spectrometry.

To confirm and begin to understand the basis for the high rate of false positives, we conducted follow-up studies. First, we repeated our analysis of the ultra-deep sequencing data with a range of thresholds that allowed less stringent criteria for mapping and base quality (Table S4). Despite this, we were not able to unmask additional mutations in the sequencing data, and the same three mutations were consistently identified. Second, we found that the three verified mutations (*NRAS*^*A18T*^, *JAK3*^*V722I*^ and *MET*^*R970C*^) were not biased toward cases with lower or higher average coverage in sequencing data when compared to those with unverified mutations (t-test, P = 0.47). Finally, we considered whether the high rate of false positives was due to inaccuracy imposed by the Typer software used to make the high confidence calls. We invited an independent clinical laboratory scientist with expertise in clinical-grade, mass spectrometry-based mutation detection to review the spectra from our initial studies. Blinded to our ultra-deep sequencing results, the manual expert reviewer dismissed four of the 14 high confidence calls as having “low reliability” (Table S3). However, this manual review did not improve the FDR, which was 79% (11 of 14) before and 80% (8 out of 10) after the review (Table S3). While some of the false positives could have been caused by adducts (especially K^+^ adducts) (Figure S1A), we point out that the PCR products had been treated with an ion-exchange resin prior to mass spectrometry to reduce this possibility. Many of the non-validated calls had no adduct potential because the mass difference separating the mutant and wild type peak was not sufficiently close to the mass shift caused by known adducts (Figure S1B–D). In those cases, cytosine deamination caused by FFPE fixation or decomposition products from other assays multiplexed into the same well may have contributed.

We explored whether the DNA concentration and “quality” (*i.e.*, from frozen or fixed material) might influence the rate of false positives in mass spectrometry-based mutation calling using two human sarcoma cell lines (RD and JR1). We prepared DNA with high (10 ng/μl) and low (0.2 ng/μl) concentration (HC and LC, respectively), and without and with exposure to 4% paraformaldehyde to model good and poor “quality” (GQ and PQ, respectively). We evaluated all four combinations (HC + GQ, LC + GQ, HC + PQ, and LC + PQ) in duplicate using the OncoCarta v1.0 and v3.0 panels, according to the manufacturer’s guidelines. As listed in Fig. 2 and Table S5, 13 high-confidence mutations, including six instances of *NRAS*^*Q61H*^, were identified in these 16 samples.

We attempted to verify the mutation calls in both cell lines using whole-exome and whole-transcriptome sequencing. Focusing on the mutated loci, whole-exome sequencing depth averaged 63× for RD cells and 76× for JR1 cells, and whole-transcriptome depth averaged 252× and 189× for RD and JR1, respectively. Among the 13 high-confidence mutations in eight different genes called using mass spectrometry, only the *NRAS*^*Q61H*^ mutation was confirmed by both whole-exome and whole-transcriptome sequencing. Consistent with our observations from actual tumor specimens, mass spectrometric mutation calls were associated with a relatively high FDR: 37.5% and 80% for good and poor quality DNA, respectively, and 54% overall (Fig. 2 and Table S5). Replicate testing, not typically utilized with mass spectrometry-based analyses as noted above, would eliminate those false-positive variants in this analysis but also might compromise sensitivity to detect true mutant alleles (Fig. 2). DNA “quality” also seemed to influence the sensitivity of mass spectrometry to detect real mutations: the *NRAS*^*Q61H*^ allele was identified in 5 of 8 replicates with high quality, and only 1 of 8 replicates with low quality DNA (Table S5). Furthermore, mass spectrometric detection of low frequency mutant alleles was always false when it indicated a mutant allele frequency of 30% or less, a finding similar to the false discovery rates in the first experiment (Fig. 1). DNA concentration did not appear to influence the FDR (50% vs. 57% in HC and LC, respectively) or sensitivity to detect a true mutation (37.5% in both in HC and LC).

Finally, we addressed whether our ultra-deep sequencing assay lacked the sensitivity to detect what we deemed to be false-positive variants, which mass spectrometry detected at an average allele frequency of 14.8% (range: 6.4–27.3%) (Table S3). To address this concern, we took advantage of the fact that two different single nucleotide variants are found in *NRAS* in the RD and Rh30 rhabdomyosarcoma cell lines (Fig. 3A). We extracted DNA from the two cell lines cells, which were either freshly frozen (FF) or fixed in 4% paraformaldehyde to mimic FFPE specimens, and we empirically determined the amount of each DNA sample that would lead to equivalently amplified DNA fragments (Fig. 3B). RD DNA was serially diluted in Rh30 DNA (*i.e.*, RD = 100%, 50%, 25%, 12.5%, 6%, 3% and 0%). We applied the same NGS approach previously applied to the tumor specimens to try to detect the RD- and Rh30-associated variants in the mixed DNA samples (average read depth after removing PCR duplicates: 1271×). The proportion of reads for each variant allele, relative to wild type, approximated the proportion of the DNA known to contain that variant (Fig. 3C). Importantly, we always detected the mutant allele by sequencing, even when the true allele frequency was as low as 3.1% in DNA extracted from either frozen or fixed samples (Fig. 3C and Table S6). This threshold is lower than the lowest variant allele called by mass spectrometry but not confirmed by our next generation sequencing (Table S3). Hence, the discordance that we observed between the variants called by mass spectrometry and DNA sequencing does not seem likely to be driven by low sensitivity of the NGS assay. In contrast to our approach in this analysis, using a small amount of starting material (*e.g.*, 1 ng genomic DNA) in a *multiplexed* PCR amplification might limit diversity in the amplified DNA used for sequencing, thereby compromising assay sensitivity.

In summary, we can draw a number of conclusions from our analysis of a relatively large panel of childhood soft tissue sarcoma specimens: First, we did find potentially actionable mutations in a small number of childhood soft tissue sarcoma specimens (3 of 52) using this commonly-employed mass spectrometry-based assay as a screen, and the putative variants were confirmed with a complementary NGS approach. The low number of actionable variants may underscore the emerging theme about the paucity of such mutations in childhood neoplasms^7^. Our findings also highlight the importance of genome-wide sequencing approaches or, when applying panel-based sequencing, utilizing those that are developed for childhood cancer. Two groups recently reported somatic mutations identified by whole exome sequencing in several types of sarcoma from adult patients^8^,^9^. Only 2 of the 169 mutations identified in those reports are represented in the OncoCarta panels. Yet, even for mutations included in the OncoCarta panels, the low sensitivity in our mass spectrometry-based studies could have limited their detection, especially in cases exposed to formaldehyde-based fixative. Beyond the low sensitivity, our analysis revealed a surprisingly high FDR in this analysis of DNA extracted from archived FFPE material from a wide range of childhood soft tissue sarcomas and other solid tumors. The high FDR may be at least partly attributed to lower “quality” DNA (*i.e.*, exposure to formaldehyde-based fixative), which also seems to compromise the aforementioned sensitivity. Hence, we suggest that researchers should be cautious about primarily using a mass spectrometry-based approach to broadly survey for mutations in DNA from FFPE material without either replicate testing or secondary validation with a complementary approach.

## Methods

### DNA Extraction

Formalin-fixed, paraffin-embedded (FFPE) tissue samples primarily representing non-rhabdomyosarcoma soft tissue sarcoma (NRSTS) were identified at the Children’s Oncology Group (COG) Biopathology Center at Nationwide Children’s Hospital. The cases had been submitted to the Biopathology Center as part of the COG D9902 Soft Tissue Sarcoma Biology/Banking study, and informed consent for specimen use was obtained prior to sample submission. The Institutional Review Board at the University of Texas Southwestern Medical Center reviewed and approved this research project (IRB # STU 102011-034); and all of the research work was accomplished in accordance with these approved guidelines.

Hematoxylin and eosin–stained slides were reviewed for each case by a pathologist to confirm diagnosis and assess tumor composition and necrosis. 50 of the 52 sample blocks were composed of >70% tumor tissue and 49 contained <30% necrosis. Between 3 and 60 15-micron sections were used to obtain 2 μg of DNA per sample. DNA was extracted from FFPE blocks of 52 NRSTS cases using the Qiagen EZ1 DNA Tissue kit (Qiagen, Valencia, CA), according to the manufacturer’s specifications. DNA concentration was measured by the PicoGreen fluorescence assay (Life Technologies, Grand Island, NY). A_260/280_ measurements were taken to assess nucleic acid purity using a NanoDrop spectrophotometer (Thermo Scientific, Wilmington, DE). The de-identified DNA samples were further analyzed in the Skapek laboratory.

### MassARRAY analysis

All 52 cases were assayed using the Sequenom MassARRAY OncoCarta v1.0 and v3.0 panels (Sequenom, San Diego, CA), according to manufacturer’s specifications, to examine 365 unique mutations in 33 cancer-related genes. Briefly, 20 ng of DNA per well was PCR amplified using predesigned, multiplexed primers in 96-well plates; treated with shrimp alkaline phosphatase; and processed with extension primers to be extended by a single base at the corresponding nucleotide mutation sites. The processed DNA was then desalted using Sequenom Clean Resin, spotted onto a SpectroCHIP array using the MassARRAY Nanodispenser, and assessed by MALDI-TOF mass spectrometry using the MassARRAY Analyzer 4. The data were analyzed in Typer 4.0.20 software (Sequenom) using the Oncomutation Report command with a frequency cutoff of 5% for the mutant peak to generate a Z-Score and OncoReport confidence level. To verify mutation calls, find calls missed by the OncoReport program, and eliminate likely false positives called by OncoReport, the spectra for each assay were manually examined and evaluated as recommended by the OncoCarta Manual Analysis Guide and Quick Guide for OncoCarta Analysis (Sequenom). In particular, the high and medium confidence calls from OncoReport were independently reviewed and scored by a clinical pathology laboratory scientist who was blinded to any additional information on the tumor specimens.

### Targeted ultra-deep sequencing

To validate the potential mutations identified by MassARRAY, we performed deep sequencing of the genomic regions containing the mutations. One case (PASNLT) could not be included in the validation set due to insufficient remaining DNA. Primers were designed to amplify approximately 150 bp segments (range 118 bp–329 bp) of DNA surrounding the mutations using SeqBuilder (DNASTAR, Madison, WI) and Primer-BLAST^10^ software (Table S7). For each reaction, 1 ng of input DNA underwent 35 cycles of PCR (20 sec denature, 15 sec anneal, 15 sec extend) using the KAPA HiFi (high fidelity) Hot Start DNA polymerase (KAPA Biosystems, Wilmington, MA). Each reaction was carried out independently, as opposed to being multiplexed. The PCR products were loaded and run on a 0.8% agarose, 0.0005% ethidium bromide gel, the bands of interest cut out with a clean, sterile razorblade on a long-wavelength (365 nm) UV box (3UV Transilluminator, UVP, Upland, CA), and extracted using the QIAquick Gel Extraction Kit (Qiagen), according to manufacturer’s specifications. Library preparation and sequencing were carried out at the McDermott Next Generation Sequencing Core. Briefly, DNA concentration was measured using a Qubit Fluorometer (Life Technologies). Library preparation was carried out using the KAPA HTP Library Preparation Kit (KAPA Biosystems) with Illumina TruSeq adapters (Illumina, San Diego, CA) at a 1:2 dilution and seven cycles of PCR. The library was quantified using a 2100 Bioanalyzer (Agilent Technologies, Santa Clara, CA) and the samples were sequenced on a HiSeq 2500 (Illumina) with 100 bp paired-end reads.

To assess the detection limits of this assay, we studied two variants in *NRAS*, one each found in either the RD or Rh30 rhabdomyosarcoma cell line. We serially diluted the RD DNA in Rh30 DNA and PCR amplified approximately 200 bp segments of DNA surrounding each sequence variant, using the same methods as described above. The products from the two reactions were gel purified, combined, and sent to the NGS Core for sequencing. To approximate the effect of formalin fixation, RD and Rh30 cells were pelleted into two tubes per cell line (1 × 10^6^ cells per tube) and either flash frozen in liquid nitrogen or fixed with 4% paraformaldehyde (PFA) for 15 minutes, then washed three times in PBS, prior to DNA extraction.

### Whole exome and transcriptome sequencing of human tumor cell lines

DNA and RNA were extracted from RD and JR1 human rhabdomyosarcoma cell lines using the Blood & Cell Culture DNA Mini Kit (Qiagen) and Trizol (Life Technologies), respectively, according to manufacturer’s specifications. Whole exome and transcriptome sequencing were carried out at the McDermott Next Generation Sequencing Core as described above, with the exception of library preparation, which was performed using the Nextera Rapid Capture Kit (Illumina) for whole exome and the TruSeq Stranded Total RNA LT Sample Prep Kit (Illumina) for whole transcriptome sequencing, according manufacturer’s specifications.

### Whole-exome, whole-transcriptome and targeted ultra-deep sequencing data analysis

All raw sequencing reads were mapped to the human reference genome (hg19) using the Burrows-Wheeler Aligner^11^. Picard was used to convert SAM files to BAM files and remove PCR duplicates (http://broadinstitute.github.io/picard/). Our analysis pipeline also realigned the sequence reads to obtain more accurate quality scores and then recalibrated the base qualities by GATK^12^ before variant calling. Variant calling was done by directly measuring the number of supporting reads for A, T, G and C on the genomic loci containing MassARRAY-identified mutations. Our computational pipeline compared variants against common polymorphisms present in the dbSNP 138 and 1000 Genomes databases, as well as COSMIC version 73, a database of cancer somatic mutations. Several gene transcript annotation databases (Consensus CDS, RefSeq, Ensembl and UCSC) were used to determine amino acid changes. All BAM files and programming scripts are available upon request.

### Statistical analysis

Based on Dziuda *et al*.^13^ and Myatt *et al*.^14^, false discovery rate (FDR) is equal to the ratio between the number of false positive mutation calls identified by NGS data and the number of all positive mutation calls identified by MassARRAY. No correction for multiple testing is needed for calculating this FDR value. In addition, unpaired T test was used for comparing average coverage between three verified mutations and other unverified mutations. P < 0.05 was considered to be statistically significant in this study.

## Additional Information

**How to cite this article**: Xu, L. *et al*. Potential pitfalls of mass spectrometry to uncover mutations in childhood soft tissue sarcoma: A report from the Children’s Oncology Group. *Sci. Rep.* **6**, 33429; doi: 10.1038/srep33429 (2016).

## Supplementary Material

Supplementary Information

## Figures and Tables

**Figure 1:**
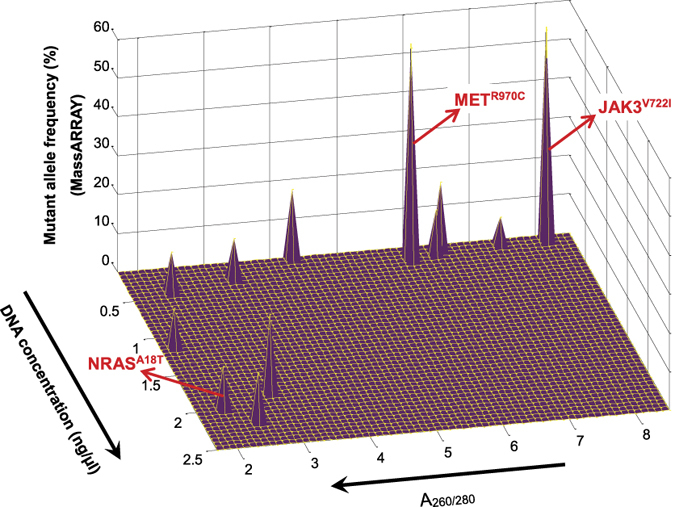
All fourteen mutations identified by MassARRAY are plotted based on their allele frequencies, DNA concentrations and A_260/280_ values. Three (*NRAS*^*A18T*^, *JAK3*^*V722I*^ and *MET*^*R970C*^) out of fourteen mutations identified by MassARRAY were validated by ultra-deep sequencing data (marked by red arrows). Note that there were twelve DNA samples that contain these fourteen mutations. For two cases in which two mutations were within the same DNA sample, the peak with lower allele frequency was buried by the one with higher allele frequency in the figure.

**Figure 2:**
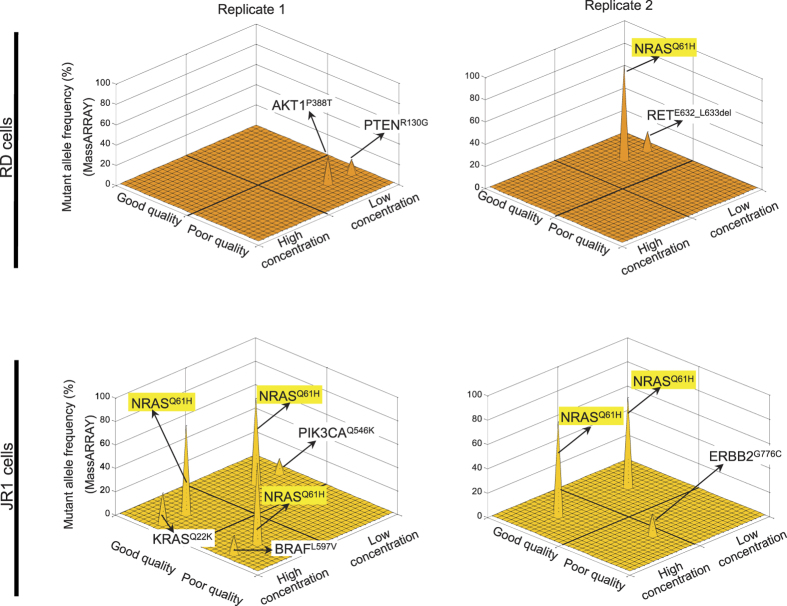
All thirteen mutations identified by MassARRAY are plotted based on their allele frequency, DNA concentration and quality in replicate analyses of RD and JR1 cells. Among all these, only *NRAS*^*Q61H*^ mutations were validated by whole-exome and whole-transcriptome sequencing (marked by yellow boxes).

**Figure 3:**
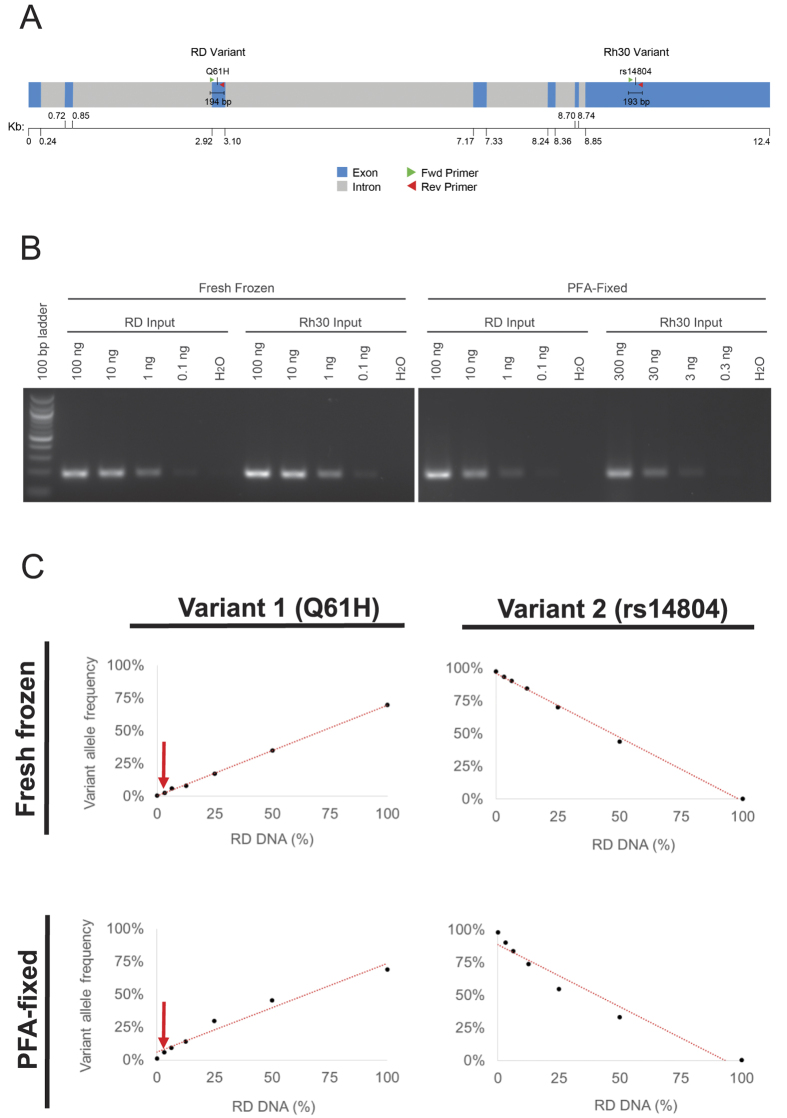
(**A**) Schematic diagram shows DNA sequence variants in the human NRAS gene in either the RD or the Rh30 rhabdomyosarcoma cell lines. Forward and reverse primers used to amplify the genomic DNA for NGS are denoted by red and green arrows. (**B**) Photograph of ethidium bromide-stained 2% agarose gel shows semi-quantitative analysis of PCR amplification of one of the products noted in (**A**), confirming equal representation of each of the two sources of input DNA used for mixing experiment. (**C**) Graphs displaying the relative frequency of calls by NGS for each variant allele in DNA samples containing DNA from RD and Rh30 cells. The variant RD allele could be detected by NGS even when present 3.1% of the total DNA (arrow).
