# Supplementary material for: Potential pitfalls of mass spectrometry to uncover mutations in childhood soft tissue sarcoma: A report from the Children’s Oncology Group
Source: Sci Rep. 2016 Sep 19;6:33429. doi: 10.1038/srep33429 (PMC5027578; doi:10.1038/srep33429)
Supplement: Supplementary Information [file srep33429-s1.pdf]

**Supplementary Information**

# Potential pitfalls of mass spectrometry to uncover mutations in childhood soft tissue sarcoma: A report from the Children's Oncology Group

Lin Xu[1,6], Raphael A. Wilson[1,6], Theodore W. Laetsch[1], Dwight Oliver[2], Sheri L. Spunt[3], Douglas S. Hawkins[4], Stephen X. Skapek[1,5]

[1]Department of Pediatrics Division of Hematology/Oncology, the [2]Department of Pathology, and [1]the Harold C. Simmons Cancer Center, University of Texas Southwestern Medical Center, and [1]the Pauline Allen Gill Center for Cancer and Blood Disorders, Children's Medical Center, Dallas, TX; [3]Department of Pediatrics, Division of Hematology/Oncology, Stanford University School of Medicine, Palo Alto, CA; [4]Division of Hematology/Oncology, Seattle Children's Hospital, Fred Hutchison Cancer Research Center, University of Washington, Seattle, WA.

**Figure S1.** False positive mimics true positive MassARRAY spectra. (A and B) Mass spectra for representative false positive calls that include mass shifts that are close to shifts that could be found from $K^+$ adduct (A) or shifts that do not align with possible salt adduct (B), which are listed in (C). Note that two of the assays contain peaks from a partially overlapping assay (*) that include 38 Da shifts due to $K^+$ adducts (#). (D) Mass spectra for the three assays in which the mutation was confirmed by targeted NGS. UEP, unextended primer peak; WT, wildtype allele peak; Mut, mutant allele peak; red arrows indicate the mass difference between the mutant peak and the wild-type peak or other neighboring peaks; purple arrows indicate the mass shift that would be caused by $K^+$ adducts on large neighboring peaks.

A

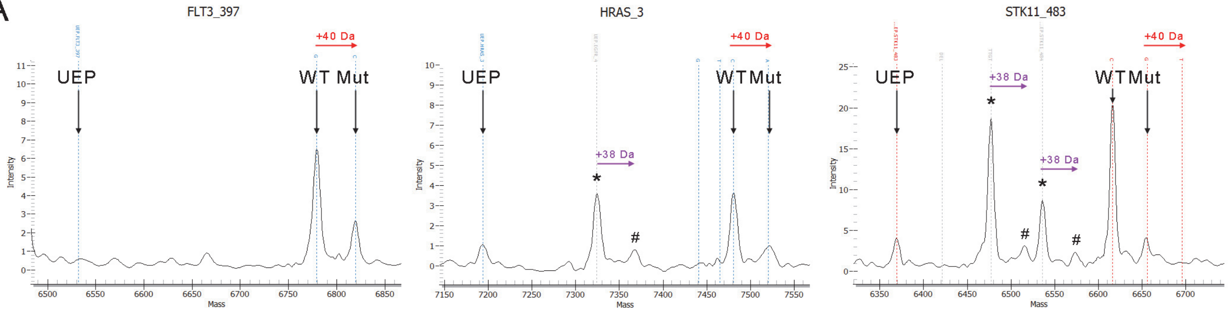


B

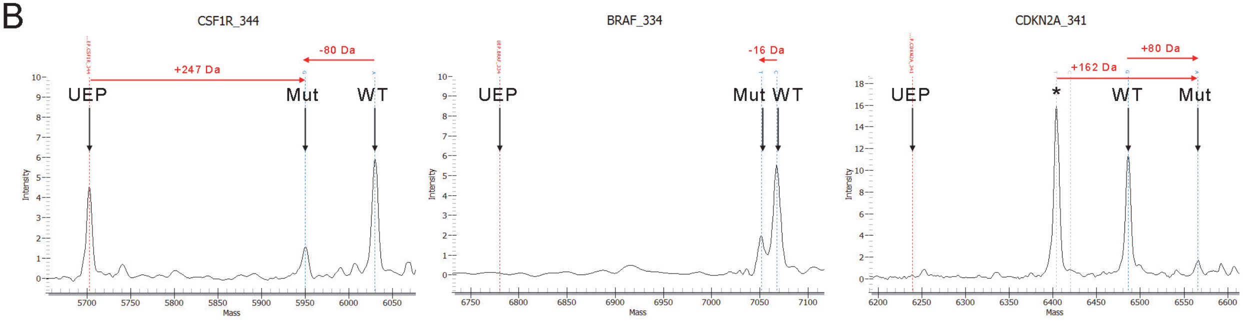


C

| Adduct | Mass Shift |
|---|---|
| Ammonia | + 17 Da |
| Sodium | + 22 Da |
| Potassium | + 38 Da |
| Carbonic Acid | + 63 Da |
| De-carboxylated 3-HPA | + 94 Da |
| Pure 3-HPA | + 138 Da |
| 2x de-carboxylated 3-HPA | + 188 Da |

D

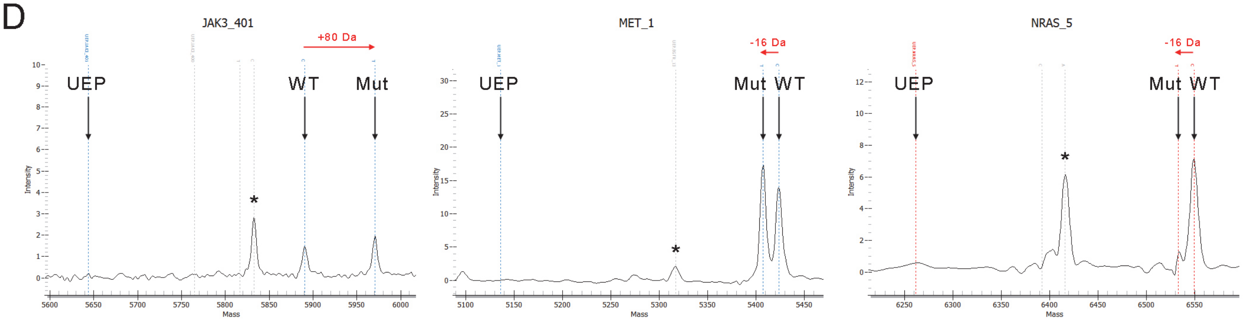

**Table S1.** A list of 60 publications using MassARRAY that were manually reviewed to determine whether samples were run in duplicate. Additional parameters and results are listed for studies that included duplicates.

| PMID | Journal | MassARRAY Duplicates? | Samples Run in Duplicate | Concordance | # of SNPs Tested | Starting Material |
|---|---|---|---|---|---|---|
| 21444862 | J Clin Oncol. 2011 Jun 10;29(17):e499-502. | No | | | | |
| 25559813 | J Clin Oncol. 2015 Apr 20;33(12):1340-7. | No | | | | |
| 21810691 | J Clin Oncol. 2011 Sep 1;29(25):3435-42. | No | | | | |
| 24470002 | J Clin Oncol. 2014 Mar 1;32(7):647-53. | No | | | | |
| 18565889 | J Clin Oncol. 2008 Jun 20;26(18):3031-7. | No | | | | |
| 18281655 | J Clin Oncol. 2008 Feb 20;26(6):842-7. | No | | | | |
| 19841321 | J Clin Oncol. 2010 Feb 1;28(4):614-9. | No | | | | |
| 18299612 | J Clin Oncol. 2008 May 1;26(13):2131-8. | Yes | 10% | Not indicated | 2 | FF |
| 24369049 | N Engl J Med. 2014 Jan 9;370(2):119-28. | No | | | | |
| 24152261 | N Engl J Med. 2013 Oct 24;369(17):1620-8. | No | | | | |
| 23406027 | N Engl J Med. 2013 Feb 14;368(7):623-32. | No | | | | |
| 22808956 | N Engl J Med. 2012 Jul 19;367(3):214-23. | No | | | | |
| 25329327 | JAMA Pediatr. 2014 Dec;168(12):1122-30. | No | | | | |
| 23695349 | JAMA. 2013 Jun 5;309(21):2232-9. | No | | | | |
| 20736472 | JAMA. 2010 Aug 25;304(8):881-9. | Yes | 1% | 99.6% | 18 | Not indicated |
| 20670955 | Cancer Res. 2010 Aug 1;70(15):6233-7. | No | | | | |
| 19010912 | Cancer Res. 2008 Nov 15;68(22):9375-83. | No | | | | |
| 17018596 | Cancer Res. 2006 Oct 1;66(19):9420-7. | No | | | | |
| 22144470 | Cancer Res. 2012 Feb 1;72(3):636-44. | No | | | | |
| 19318546 | Cancer Res. 2009 Apr 15;69(8):3605-13. | Yes | 22% | >95% | 6 | FF |
| 19567675 | Cancer Res. 2009 Jul 15;69(14):5970-7. | Yes | 23% | >95% | 15 | Not indicated |
| 19738052 | Cancer Res. 2009 Sep 15;69(18):7459-65. | No | | | | |
| 21368091 | Cancer Res. 2011 Apr 15;71(8):3000-8. | No | | | | |
| 25929848 | Cancer Discov. 2015 Jun;5(6):610-21. | Yes | 100% | Not indicated | 92 | Not indicated |
| 25971939 | Cancer Discov. 2015 Aug;5(8):842-9. | No | | | | |
| 24634376 | Clin Cancer Res. 2014 May 1;20(9):2326-37. | No | | | | |
| 23092874 | Clin Cancer Res. 2012 Dec 15;18(24):6784-91. | No | | | | |
| 24449825 | Clin Cancer Res. 2014 Mar 15;20(6):1676-86. | No | | | | |
| 20959405 | Clin Cancer Res. 2010 Nov 1;16(21):5339-50. | Yes | Not indicated | Not indicated | 79 | FF |
| 22228640 | Clin Cancer Res. 2012 Feb 15;18(4):1167-76. | Yes | 100% | Not indicated | 92 | FFPE |
| 25589623 | Clin Cancer Res. 2015 Apr 1;21(7):1591-601. | Yes | 7% | Not indicated | 4 | FF |
| 21242121 | Clin Cancer Res. 2011 Apr 15;17(8):2410-6. | No | | | | |
| 24980946 | Mol Cancer Ther. 2014 Sep;13(9):2226-37. | No | | | | |
| 22383533 | Mol Cancer Ther. 2012 Apr;11(4):888-97. | No | | | | |
| 23371856 | Mol Cancer Ther. 2013 Feb;12(2):220-9. | Yes | 100% | Not indicated | 92 | Not indicated |
| 25833835 | Mol Cancer Ther. 2015 Jun;14(6):1466-75. | No | | | | |
| 19226183 | PLoS Med. 2009 Feb 17;6(2):e1000029. | No | | | | |
| 25647612 | PLoS Med. 2015 Feb 3;12(2):e1001782. | No | | | | |
| 20617178 | PLoS Pathog. 2010 Jul 1;6:e1000979. | No | | | | |
| 21852947 | PLoS Pathog. 2011 Aug;7(8):e1002174. | No | | | | |
| 17803357 | PLoS Biol. 2007 Sep;5(9):e236. | No | | | | |
| 16248677 | PLoS Biol. 2005 Nov;3(11):e378. | No | | | | |
| 23359636 | PLoS Genet. 2013;9(1):e1003183. | No | | | | |
| 23028341 | PLoS Genet. 2012 Sep;8(9):e1002916. | Yes | 1-4% | 99.8% | 10 | FF |
| 20072603 | PLoS Genet. 2010 Jan 8;6(1):e1000806. | Yes | Not indicated | 99.8% | 9 | FF |
| 20169178 | PLoS Genet. 2010 Feb 12;6(2):e1000843. | No | | | | |
| 21124955 | PLoS Genet. 2010 Nov 18;6(11):e1001213. | Yes | 10% | 99.5% | 27 | FF |
| 17677000 | PLoS Genet. 2007 Jul;3(7):e120. | Yes | 5% | 99.8% | 53 | FF |
| 17925536 | J Natl Cancer Inst. 2007 Oct 17;99(20):1525-33 | No | | | | |
| 20484103 | J Natl Cancer Inst. 2010 Jul 7;102(13):972-81. | Yes | 2% | 99.6% | 7 | FF |
| 23411593 | J Natl Cancer Inst. 2013 Apr 17;105(8):573-9. | Yes | 2% | 99.6% | 2 | FF |

| | | | | | | |
|---|---|---|---|---|---|---|
| 22565307 | J Clin Invest. 2012 Jun;122(6):2316-25. | No | | | | |
| 22182842 | J Clin Invest. 2012 Jan;122(1):205-17. | No | | | | |
| 21804190 | J Clin Invest. 2011 Sep;121(9):3635-44. | No | | | | |
| 24244628 | PLoS One. 2013 Nov 14;8(11):e80132. | No | | | | |
| 24945726 | PLoS One. 2014 Jun 19;9(6):e100515. | No | | | | |
| 25121767 | PLoS One. 2014 Aug 14;9(8):e104966. | No | | | | |
| 23717594 | PLoS One. 2013 May 22;8(5):e64310. | Yes | 5% | 100% | 1 | FF |
| 24999991 | PLoS One. 2014 Jul 7;9(7):e99409. | No | | | | |
| 24762746 | PLoS One. 2014 Apr 24;9(4):e95080. | No | | | | |

| |
|---|
| All samples run in duplicate |
| Low (or uncertain) % of samples run in duplicate |
| No mention of MassARRAY replicates |

**Table S2.** A list of 52 tumor specimens used in this study, which represents 18 different types of pediatric sarcoma or related soft tissue neoplasm

| Sample | Type |
|---|---|
| PAPZHZ | undifferentiated sarcoma (UDS) |
| PASNLT | Fibrosarcoma |
| PASFLR | Epithelioid Sarcoma |
| PANFBM | Synovial Sarcoma |
| PASHNI | Synovial Sarcoma |
| PARLCI | Synovial Sarcoma |
| PANREV | Pleomorphic Sarcoma |
| PATRVC | Epithelioid Sarcoma |
| PATWAW | Desmoplastic small round cell tumor (DSRCT) |
| PARHGI | Synovial Sarcoma |
| PANLJD | Synovial Sarcoma |
| PANYNB | Malignant peripheral nerve sheath tumors (MPNST) |
| PATPTL | Soft tissue sarcoma Unclassified |
| PARCSZ | Synovial Sarcoma |
| PATCFN | Epithelioid Sarcoma |
| PASARX | Malignant peripheral nerve sheath tumors (MPNST) |
| PASAAK | Dermatofibrosarcoma Protuberans |
| PATJPV | Soft tissue sarcoma Unclassified |
| PASDWC | Synovial Sarcoma |
| PAPXLN | Undifferentiated embryonal sarcoma of the liver (UESL) |
| PATDRA | Soft tissue sarcoma Unclassified |
| PASBFM | Malignant peripheral nerve sheath tumors (MPNST) |
| PATZJG | Myxoid Liposarcoma |
| PANYEP | Desmoplastic small round cell tumor (DSRCT) |
| PATTXI | Malignant peripheral nerve sheath tumors (MPNST) |
| PATCFF | Synovial Sarcoma |
| PASRNG | Synovial Sarcoma |
| PASIZZ | Soft tissue sarcoma Unclassified |
| PASMXP | Malignant peripheral nerve sheath tumors (MPNST) |
| PAMHMJ | Fibromyxoid Tumor |
| PASJEH | Malignant peripheral nerve sheath tumors (MPNST) |
| PATWZS | Synovial Sarcoma |
| PAMLLB | Infantile Fibrosarcoma |
| PASUTY | undifferentiated sarcoma (UDS) |
| PAPRMD | undifferentiated sarcoma (UDS) |
| PATGTB | Soft tissue sarcoma Unclassified |
| PASTRM | Synovial Sarcoma |
| PARMAJ | Synovial Sarcoma |
| PASVYN | Synovial Sarcoma |
| PAPKTY | Alveolar Soft Part Sarcoma |
| PALPNJ | Synovial Sarcoma |
| PAPWGG | Undifferentiated embryonal sarcoma of the liver (UESL) |
| PARFNC | Malignant peripheral nerve sheath tumors (MPNST) |

| | |
|---|---|
| PANYDZ | Soft tissue sarcoma Unclassified |
| PATISR | Liposarcoma |
| PASLWE | Neurofibroma |
| PATJAC | Myofibroblastic |
| PATYZM | Soft tissue sarcoma Unclassified |
| PATJRF | Desmoid-type Fibromatosis |
| PATLDX | Myxoid Liposarcoma |
| PAREBM | Liposarcoma |
| PARVMN | Malignant peripheral nerve sheath tumors (MPNST) |

**Table S3.** A list of 15 high-confidence mutations identified by MassARRAY. Three mutations highlighed by green color are the ones validated by ultra-deep sequencing data.

| | Type | Mutation | Evaluation by MassARRAY software (Confidence level) | Independent manual review by a experienced scientist | MassARRAY (% mutant) | NGS platform, Reads (All) | NGS platform, Reads (Mut) | NGS platform, % Mutation |
|---|---|---|---|---|---|---|---|---|
| PANYEP | DSRCT | CSF1R_Y969H | High | high reliability | 22.3 | 827 | 4 | 0.48 |
| PAREBM | Liposarcoma 2 | JAK3_V722I | High | high reliability | 54.7 | 2114 | 960 | 45.41 |
| PARFNC | MPNST 1 | MET_R970C | High | high reliability | 55.3 | 866 | 519 | 59.93 |
| PAPZHZ | UDS 1 | CDKN2A_H83Y | High | low reliability | 10.4 | 2379 | 15 | 0.63 |
| PAPKTY | Alveolar Soft Part Sarcoma | STK11_G196V | High | high reliability | 18.4 | 309 | 0 | 0.00 |
| PASAAK | DFSP | STK11_P281L | High | high reliability | 6.4 | 446 | 0 | 0.00 |
| PASAAK | DFSP | BRAF_V471F | High | high reliability | 12.9 | 1221 | 0 | 0.00 |
| PANYEP | DSRCT | BRAF_V471F | High | high reliability | 20.7 | 1170 | 0 | 0.00 |
| PAMLLB | Infantile Fibrosarcoma | BRAF_V600M | High | low reliability | 11 | 689 | 2 | 0.29 |
| PATISR | Liposarcoma 1 | FLT3_I836M | High | high reliability | 18 | 835 | 1 | 0.12 |
| PATJAC | Myofibroblastic | FLT3_I836M | High | high reliability | 7.6 | 933 | 0 | 0.00 |
| PANREV | Pleomorphic Sarcoma | NRAS_A18T | High | low reliability | 11.3 | 1950 | 62 | 3.18 |
| PARCSZ | Synovial Sarcoma 2 | FLT3_I836M | High | low reliability | 11.1 | 4733 | 1 | 0.02 |
| PANYDZ | STS Unclassified | FLT3_I836M | High | high reliability | 11.9 | 684 | 0 | 0.00 |
| PASNLT | Fibrosarcoma | HRAS_G13C | High | low reliability | 27.3 | No DNA | No DNA | No DNA |

**Table S4.** The analyses of the ultra-deep sequencing data repeated using a range of thresholds for mapping and base quality.

| | | | | | Base quality >5 | | | | | | | | |
|---|---|---|---|---|---|---|---|---|---|---|---|---|---|
| | | | | | Mapping quality >10 | | | Mapping quality >25 | | | Mapping quality >40 | | |
| | Type | Mutation | OncoReport Confidence | MassARRAY (% mutant) | Reads (All) | Reads (Mut) | % Mutation | Reads (All) | Reads (Mut) | % Mutation | Reads (All) | Reads (Mut) | % Mutation |
| PANYEP | DSRCT | CSF1R_Y969H | High | 22.3 | 833 | 4 | 0.48 | 827 | 4 | 0.48 | 676 | 3 | 0.44 |
| PAREBM | Liposarcoma | JAK3_V722I | High | 54.7 | 2163 | 986 | 45.58 | 2114 | 960 | 45.41 | 744 | 341 | 45.83 |
| PARFNC | MPNST | MET_R970C | High | 55.3 | 930 | 547 | 58.82 | 866 | 519 | 59.93 | 514 | 353 | 68.68 |
| PAPZHZ | UDS | CDKN2A_H83Y | High | 10.4 | 2402 | 15 | 0.62 | 2379 | 15 | 0.63 | 1032 | 10 | 0.97 |
| PAPKTY | Alveolar Soft Part Sarcoma | STK11_G196V | High | 18.4 | 310 | 0 | 0.00 | 309 | 0 | 0.00 | 272 | 0 | 0.00 |
| PASAAK | DFSP | STK11_P281L | High | 6.4 | 447 | 0 | 0.00 | 446 | 0 | 0.00 | 259 | 0 | 0.00 |
| PASAAK | DFSP | BRAF_V471F | High | 12.9 | 1224 | 0 | 0.00 | 1221 | 0 | 0.00 | 905 | 0 | 0.00 |
| PANYEP | DSRCT | BRAF_V471F | High | 20.7 | 1178 | 0 | 0.00 | 1170 | 0 | 0.00 | 726 | 0 | 0.00 |
| PAMLLB | Fibrosarcoma | BRAF_V600M | High | 11 | 689 | 2 | 0.29 | 689 | 2 | 0.29 | 608 | 2 | 0.33 |
| PATISR | Liposarcoma | FLT3_I836M | High | 18 | 835 | 1 | 0.12 | 835 | 1 | 0.12 | 799 | 1 | 0.13 |
| PATJAC | Myofibroblastic | FLT3_I836M | High | 7.6 | 933 | 0 | 0.00 | 933 | 0 | 0.00 | 892 | 0 | 0.00 |
| PANREV | Pleomorphic Sarcoma | NRAS_A18T | High | 11.3 | 1950 | 62 | 3.18 | 1950 | 62 | 3.18 | 1803 | 58 | 3.22 |
| PARCSZ | Synovial Sarcoma | FLT3_I836M | High | 11.1 | 4733 | 1 | 0.02 | 4733 | 1 | 0.02 | 4468 | 1 | 0.02 |
| PANYDZ | STS Unclassified | FLT3_I836M | High | 11.9 | 684 | 0 | 0.00 | 684 | 0 | 0.00 | 539 | 0 | 0.00 |

| | | | | | Base quality >10 | | | | | | | | |
|---|---|---|---|---|---|---|---|---|---|---|---|---|---|
| | | | | | Mapping quality >10 | | | Mapping quality >25 | | | Mapping quality >40 | | |
| | Type | Mutation | OncoReport Confidence | MassARRAY (% mutant) | Reads (All) | Reads (Mut) | % Mutation | Reads (All) | Reads (Mut) | % Mutation | Reads (All) | Reads (Mut) | % Mutation |
| PANYEP | DSRCT | CSF1R_Y969H | High | 22.3 | 818 | 3 | 0.37 | 812 | 3 | 0.37 | 666 | 3 | 0.45 |
| PAREBM | Liposarcoma | JAK3_V722I | High | 54.7 | 2143 | 980 | 45.73 | 2096 | 954 | 45.52 | 740 | 339 | 45.81 |
| PARFNC | MPNST | MET_R970C | High | 55.3 | 911 | 531 | 58.29 | 847 | 503 | 59.39 | 504 | 344 | 68.25 |
| PAPZHZ | UDS | CDKN2A_H83Y | High | 10.4 | 2375 | 15 | 0.63 | 2352 | 15 | 0.64 | 1025 | 10 | 0.98 |
| PAPKTY | Alveolar Soft Part Sarcoma | STK11_G196V | High | 18.4 | 305 | 0 | 0.00 | 304 | 0 | 0.00 | 270 | 0 | 0.00 |
| PASAAK | DFSP | STK11_P281L | High | 6.4 | 444 | 0 | 0.00 | 443 | 0 | 0.00 | 257 | 0 | 0.00 |
| PASAAK | DFSP | BRAF_V471F | High | 12.9 | 1220 | 0 | 0.00 | 1217 | 0 | 0.00 | 903 | 0 | 0.00 |
| PANYEP | DSRCT | BRAF_V471F | High | 20.7 | 1175 | 0 | 0.00 | 1167 | 0 | 0.00 | 723 | 0 | 0.00 |
| PAMLLB | Fibrosarcoma | BRAF_V600M | High | 11 | 681 | 2 | 0.29 | 681 | 2 | 0.29 | 603 | 2 | 0.33 |
| PATISR | Liposarcoma | FLT3_I836M | High | 18 | 815 | 1 | 0.12 | 815 | 1 | 0.12 | 780 | 1 | 0.13 |
| PATJAC | Myofibroblastic | FLT3_I836M | High | 7.6 | 900 | 0 | 0.00 | 900 | 0 | 0.00 | 859 | 0 | 0.00 |

| | | | | | | | | | | | | | |
|---|---|---|---|---|---|---|---|---|---|---|---|---|---|
| PANREV | Pleomorphic Sarcoma | NRAS_A18T | High | 11.3 | 1881 | 62 | 3.30 | 1881 | 62 | 3.30 | 1742 | 58 | 3.33 |
| PARCSZ | Synovial Sarcoma | FLT3_I836M | High | 11.1 | 4717 | 1 | 0.02 | 4717 | 1 | 0.02 | 4453 | 1 | 0.02 |
| PANYDZ | STS Unclassified | FLT3_I836M | High | 11.9 | 675 | 0 | 0.00 | 675 | 0 | 0.00 | 531 | 0 | 0.00 |

| | | | | | Base quality >20 | | | | | | | | |
|---|---|---|---|---|---|---|---|---|---|---|---|---|---|
| | | | | | Mapping quality >10 | | | Mapping quality >25 | | | Mapping quality >40 | | |
| | Type | Mutation | OncoReport Confidence | MassARRAY (% mutant) | Reads (All) | Reads (Mut) | % Mutation | Reads (All) | Reads (Mut) | % Mutation | Reads (All) | Reads (Mut) | % Mutation |
| PANYEP | DSRCT | CSF1R_Y969H | High | 22.3 | 647 | 1 | 0.15 | 643 | 1 | 0.16 | 535 | 1 | 0.19 |
| PAREBM | Liposarcoma | JAK3_V722I | High | 54.7 | 1569 | 897 | 57.17 | 1541 | 875 | 56.78 | 523 | 310 | 59.27 |
| PARFNC | MPNST | MET_R970C | High | 55.3 | 428 | 251 | 58.64 | 415 | 244 | 58.80 | 286 | 190 | 66.43 |
| PAPZHZ | UDS | CDKN2A_H83Y | High | 10.4 | 2060 | 7 | 0.34 | 2038 | 7 | 0.34 | 888 | 4 | 0.45 |
| PAPKTY | Alveolar Soft Part Sarcoma | STK11_G196V | High | 18.4 | 272 | 0 | 0.00 | 271 | 0 | 0.00 | 247 | 0 | 0.00 |
| PASAAK | DFSP | STK11_P281L | High | 6.4 | 370 | 0 | 0.00 | 369 | 0 | 0.00 | 217 | 0 | 0.00 |
| PASAAK | DFSP | BRAF_V471F | High | 12.9 | 1149 | 0 | 0.00 | 1148 | 0 | 0.00 | 857 | 0 | 0.00 |
| PANYEP | DSRCT | BRAF_V471F | High | 20.7 | 1014 | 0 | 0.00 | 1009 | 0 | 0.00 | 644 | 0 | 0.00 |
| PAMLLB | Fibrosarcoma | BRAF_V600M | High | 11 | 414 | 1 | 0.24 | 414 | 1 | 0.24 | 371 | 1 | 0.27 |
| PATISR | Liposarcoma | FLT3_I836M | High | 18 | 588 | 0 | 0.00 | 588 | 0 | 0.00 | 568 | 0 | 0.00 |
| PATJAC | Myofibroblastic | FLT3_I836M | High | 7.6 | 696 | 0 | 0.00 | 696 | 0 | 0.00 | 669 | 0 | 0.00 |
| PANREV | Pleomorphic Sarcoma | NRAS_A18T | High | 11.3 | 1346 | 56 | 4.16 | 1346 | 56 | 4.16 | 1255 | 54 | 4.30 |
| PARCSZ | Synovial Sarcoma | FLT3_I836M | High | 11.1 | 4502 | 1 | 0.02 | 4502 | 1 | 0.02 | 4255 | 1 | 0.02 |
| PANYDZ | STS Unclassified | FLT3_I836M | High | 11.9 | 599 | 0 | 0.00 | 599 | 0 | 0.00 | 474 | 0 | 0.00 |

**Table S5.** Mass spectrometry-based mutation calling results using two human sarcoma cell lines (RD and JR1)

*MassARRAY allele frequencies of 13 mutations are shown.*
*All blank regions indicate that no mutation was detected by MassARRAY.*
*Whole exome and transcriptome sequencing data shows that both cell lines harbor NRAS (Q61H) mutations.*

**RD**

| | NRAS (Q61H) | RET (E632_L633del) | AKT1 (P388T) | PTEN (R130G) |
|---|---|---|---|---|
| High concentration, Good quality DNA, replicate 1 | | | | |
| High concentration, Good quality DNA, replicate 2 | | | | |
| Low concentration, Good quality DNA, replicate 1 | | | | |
| Low concentration, Good quality DNA, replicate 2 | 84% | 16% | | |
| High concentration, Poor quality DNA, replicate 1 | | | | |
| High concentration, Poor quality DNA, replicate 2 | | | | |
| Low concentration, Poor quality DNA, replicate 1 | | | 26% | 13% |
| Low concentration, Poor quality DNA, replicate 2 | | | | |

**JR1**

| | NRAS (Q61H) | KRAS (Q22K) | PIK3CA (Q546K) | BRAF (L597V) | ERBB2 (G776C) |
|---|---|---|---|---|---|
| High concentration, Good quality DNA, replicate 1 | 75% | 26% | | | |
| High concentration, Good quality DNA, replicate 2 | 77% | | | | |
| Low concentration, Good quality DNA, replicate 1 | 72% | | 12% | | |
| Low concentration, Good quality DNA, replicate 2 | 73% | | | | |
| High concentration, Poor quality DNA, replicate 1 | 68% | | | 15% | |
| High concentration, Poor quality DNA, replicate 2 | | | | | 17% |
| Low concentration, Poor quality DNA, replicate 1 | | | | | |
| Low concentration, Poor quality DNA, replicate 2 | | | | | |

**Table S6.** Results of ultra-deep NGS analysis of mixed genomic DNA from RD and Rh30 cell lines. Yellow bar highlights detection of RD-specific variant present at lowest tested concentration.

| Sequencing ID | Tissue Type | % RD DNA | % Rh30 DNA | Variant 1 (chr1:115,256,528), allele frequency (alt reads / total reads) | Variant 2 (chr1:115,249,843), allele frequency (alt reads / total reads) |
|---|---|---|---|---|---|
| RD FF 1 1 | Fresh Frozen | 100% | 0% | 70% (971/1392) | 0% (4/1368) |
| RD FF 1 2 | Fresh Frozen | 50% | 50% | 35% (460/1320) | 44% (627/1426) |
| RD FF 1 4 | Fresh Frozen | 25% | 75% | 17% (230/1329) | 70% (974/1386) |
| RD FF 1 8 | Fresh Frozen | 12.5% | 87.5% | 8% (116/1471) | 84% (1203/1425) |
| RD FF 1 16 | Fresh Frozen | 6.25% | 93.75% | 6% (77/1331) | 90% (992/1100) |
| RD FF 1 32 | Fresh Frozen | 3.125% | 96.88% | 2% (35/1476) | 93% (1153/1235) |
| RD FF Zero | Fresh Frozen | 0% | 100% | 0% (5/1415) | 97% (1265/1298) |
| RD PFA-Fixed 1 1 | PFA-Fixed | 100% | 0% | 69% (875/1269) | 1% (7/1264) |
| RD PFA-Fixed 1 2 | PFA-Fixed | 50% | 50% | 45% (533/1174) | 33% (360/1083) |
| RD PFA-Fixed 1 4 | PFA-Fixed | 25% | 75% | 30% (334/1131) | 55% (652/1189) |
| RD PFA-Fixed 1 8 | PFA-Fixed | 12.5% | 87.5% | 14% (170/1201) | 74% (999/1355) |
| RD PFA-Fixed 1 16 | PFA-Fixed | 6.25% | 93.75% | 9% (110/1179) | 84% (914/1094) |
| RD PFA-Fixed 1 32 | PFA-Fixed | 3.125% | 96.88% | 6% (72/1271) | 90% (1039/1154) |
| RD PFA-Fixed Zero | PFA-Fixed | 0% | 100% | 1% (12/1203) | 98% (1028/1049) |

**Table S7.** Primers used to amplify DNA surrounding mutation sites for ultra-deep sequencing.

| **Mutation** | **Forward Primer** | **Reverse Primer** |
|---|---|---|
| ABL1_E255K | GCCCCAAAGCGCAACAAGC | GCACCCCCGGCAGTCCC |
| ABL1_G250E | GCCCCAAAGCGCAACAAGC | GCACCCCCGGCAGTCCC |
| AKT1_rs11555435 | GCGTGCTGGCCGTGCTTT | CAGTCCACCGCCGCCTCA |
| APC_E1306* | CAGCTGAAGATGAAATAGGATGTA | GACACTGCTGGAACTTCGCTCACA |
| BRAF_V471F | AGACGGGACTCGAGTGATGATTGG | TTTATTGATGCGAACAGTGA |
| BRAF_L597S/R/Q/V | TTCCTTTACTTACTACACCTCAGA | AATCAGTGGAAAAATAGCCTCAAT |
| BRAF_V600M | TTCCTTTACTTACTACACCTCAGA | AATCAGTGGAAAAATAGCCTCAAT |
| CDKN2A_H83Y | GCGCGGAGCCCAACTGC | CAGGTACCGTGCGACATC |
| CSF1R_Y969H | GCAGAAGCGGTGGCAGCG | TGAGTGAAATGACCGAAGGCAGAG |
| EGFR_G719D | ACCCTTGTCTCTGTGTTCTTGTCC | CCCCACCAGACCATGAGAGGC |
| EGFR_L747_T751>P | GGGGTGCATCGCTGGTAACATC | GGAAAGACATAGAAAGTGAACATT |
| EGFR_M766_A767insAI | GGTCCATGTGCCCCTCC | CCCTTCCCTGATTACCTTTGCGAT |
| EGFR_N771_P772>SVDNR | GGTCCATGTGCCCCTCC | CCCTTCCCTGATTACCTTTGCGAT |
| EGFR_H773_V774insH | GGTCCATGTGCCCCTCC | CCCTTCCCTGATTACCTTTGCGAT |
| EGFR_H773_V774insNPH | GGTCCATGTGCCCCTCC | CCCTTCCCTGATTACCTTTGCGAT |
| ERBB2_S779_P780insVGS | TGTGGTCTCCCATACCCTCTCAGC | GCGTCCGCGGTTTTCCC |
| FGFR3_A391E | TGTCTTTGCAGCCGAGGAGGAG | TGTGCACGGTGGGGGAGC |
| FLT3_I836M | TTGCACTCCAGGATAATACACAT | TAACGACACAACACAAAATAGCCG |
| HRAS_G13C | AGGCCCCTGAGGAGCGATGAC | GGGGTCGTATTCGTCCACAAAATG |
| HRAS_Q61L | CCGGAAGCAGGTGGTCATTGAT | GCCAGCCTCACGGGGTTCA |
| JAK3_V722I | CCGGGAGGCGCAGACACTTA | CGATGCCGGGTGAGGGG |
| MET_R970C | GTTCTGGGCACTGGGTCAAAG | TGGGCTTACACTTCGGGCACTTA |
| NRAS_A18T | GGCTCGCCAATTAACCCTGAT | GCTACCACTGGGCCTCACCTCTAT |
| NRAS_Q61H | AGCATTGCATTCCCTGTGGTTTTT | TTCGCCTGTCCTCATGTATTGGTC |
| NRAS_rs14804 | TTTGAGAGCAGATGCCATTTTGTA | TATCAGTAAGCTAGGGGAAACA |
| PDGFRA_D1071N | TCCCTCCTCCAGCTCGCAGAC | CAGAGGTGGCCCCAGAAGTG |
| STK11_G196V | CTGCCCGCAGGTACTTCTGTCAG | GGTCCGGCAGGTGTCGTCC |
| STK11_P281L | CTTCGAAGGGGACAACATCTACAA | CCTGACACCCCCAACCCTACATT |
